# Stability of a Lipase Extracted from Seeds of *Pachira aquatica* in Commercial Detergents and Application Tests in Poultry Wastewater Pretreatment and Fat Particle Hydrolysis

**DOI:** 10.1155/2013/324061

**Published:** 2013-12-23

**Authors:** Patrícia Peres Polizelli, Fernanda Dell Antonio Facchini, Gustavo Orlando Bonilla-Rodriguez

**Affiliations:** Department of Chemistry and Environmental Sciences, IBILCE-UNESP, State University of São Paulo, Rua Cristóvão Colombo 2265, 15054-000 São José do Rio Preto, SP, Brazil

## Abstract

A protein extract containing a plant lipase from oleaginous seeds of *Pachira aquatica* was tested using soybean oil, wastewater from a poultry processing plant, and beef fat particles as substrate. The hydrolysis experiments were carried out at a temperature of 40°C, an incubation time of 90 minutes, and pH 8.0-9.0. The enzyme had the best stability at pH 9.0 and showed good stability in the alkaline range. It was found that *P. aquatica* lipase was stable in the presence of some commercial laundry detergent formulations, and it retained full activity up to 0.35% in hydrogen peroxide, despite losing activity at higher concentrations. Concerning wastewater, the lipase increased free fatty acids release by 7.4 times and promoted the hydrolysis of approximately 10% of the fats, suggesting that it could be included in a pretreatment stage, especially for vegetable oil degradation.

## 1. Introduction

Lipases (triacylglycerol lipases, E.C. 3.1.1.3) are enzymes that catalyze the cleavage of carboxyl ester bonds present in acylglycerols with the subsequent release of fatty acids and glycerol. They are particularly important because they specifically hydrolyze oils and fats, an interesting ability for different industrial applications [1]. These enzymes have become more and more prominent on the enzyme biotechnology scenario due to their versatility for hydrolysis and synthesis and for their catalytic reactions often being chemoselective, regionselective, or enantioselective [2].

Lipases can be obtained from animals (pancreatic, hepatic, and gastric sources), microorganisms (bacterial, fungal, and yeast), or plants, with variations in their catalytic properties [3]. Nevertheless, recently, seed lipases have been the focus of increasing attention as biocatalysts. In some cases, these enzymes present advantages over animal and microbial lipases due to some very interesting features such as specificity, low cost, availability, and easily purification, representing a great alternative for potential commercial use as industrial enzymes [4–6].

The participation of lipases in the worldwide enzyme industry market has grown significantly and it includes a wide range of applications in many sectors such as food, pharmaceutical, fine chemical, oil chemical, and detergent industries as well as in biodiesel and wastewater treatment [7–9]. One interesting application of lipases is their use in oil chemical industries, reducing energy expenses and minimizing heat degradation of compounds in comparison to traditional chemical processes [7]. Another potential application is in wastewater treatment where several aerobic and anaerobic processes are used. However, it is necessary to reduce fats (which in high concentration can cause sludge flotation and/or the development of sludge with different physical characteristics and/or poor activity), oil, and protein concentration in order to enable biological treatment to proceed without any inhibition during the reduction of organic matter in wastewater [10–12]. The previous researchers suggested that the lipase application in pretreatment to partially hydrolyze fat particles could accelerate the anaerobic treatment of slaughterhouse wastewater and several research studies have been done on the treatment of oily wastes using lipases [13–18].

In waste treatment, the use of low-cost enzymatic preparations is vital, since the use of high-cost commercial enzymatic preparations would make the pretreatment procedure not feasible [11].

The characteristics displayed by lipases have accounted for a marked increase in their industrial use over the last two decades. However, their single biggest market is detergent formulations. The functional importance of lipases in detergent industries is to remove fatty residues in laundry and dishwashers, for cleaning clogged drains, and for avoiding pipes' clogging [4, 19–21]. Some studies have been done adding lipases to detergent formulations or adding these detergents to lipase assays to evaluate enzyme stability [4, 9, 20]. A variety of detergents were evaluated for their efficiency in removing olive oil from cotton fabrics with and without lipase; removal was higher (7–12%) in the presence of a lipase for all the tested detergents [20]. Similar results were reported for lipases from *Ralstonia pickettii* [22].

The application of a pretreatment process to hydrolyze and dissolve fats may improve the biological degradation of fatty wastewater, accelerating the process by decreasing the fat adsorption to the surface of the anaerobic sludge and not limiting the transport of the soluble substrate to the biomass [10, 23]. Beef fat particles represent a regional environmental concern due to the large number of slaughterhouses located in the State of São Paulo and nearby. Beef fat was chosen in this work because it demonstrated better results than pork fat as described by Masse et al. [13] possibly due to its different fatty acid composition.

The present work reports the effects and the characterization of a plant lipase extracted from *P. aquatica,* able to hydrolyze soybean oil and fats from poultry wastewater as well as its stability under different commercial detergents.

## 2. Materials and Methods

### 2.1. Enzyme Extraction

The seeds were collected from *Pachira aquatica* trees at UNESP (São Paulo State University), in São José do Rio Preto, SP, Brazil. The oilseeds were washed, peeled, and extracted by homogenization in a food processor with a solution described by Polizelli et al. [4], containing 3 mM DTT (dithiothreitol), 1 mM EDTA, 10 mM sodium metabisulfite, and 50 mM Tris buffer (pH 8.0) at 25°C. The samples were clarified by centrifugation at 9,000 ×g for 40 min, at 4°C using a *Jouan* CR3i refrigerated centrifuge, (4°C) and the supernatant was concentrated and stored in a freezer (Thermo Scientific) at −80°C. The Tris buffers were prepared at room temperature but their pH was calculated for the temperature of the assay, using the temperature factor ΔpKa/°C −0.031 [24].

### 2.2. Lipase Assay and Protein Determination

The lipolytic activity was measured by titration as already described [4]. Free fatty acids concentration was also determined by a titration method, and a standard curve was prepared with linoleic acid to determine the release of fatty acids during hydrolysis. The reaction solution contained 50% soybean oil, 31.3% 30 mM Tris buffer (pH 8.0), 10.15% Triton X-100, 0.002% of 10 mM CaCl_2_, and 9.6% crude extract. The reaction was stopped by adding acetone : ethanol (1 : 1), and the released fatty acids were titrated with 50 mM KOH in the presence of phenolphthalein as indicator. Protein concentrations were measured using the dye binding method [25] and using BSA (bovine serum albumin) as a standard protein.

### 2.3. Lipase Characterization

The influence of pH varying from 3.0 to 10.0 and temperature from 25 to 60°C in lipase activity was evaluated using soybean oil and poultry wastewater as enzymatic substrates. Soybean oil was chosen in this work because it is the cheapest and the most common cooking oil in Brazil, and therefore it contributes to water and soil pollution as a domestic and industrial wastewater. The enzymatic activity was analyzed as described above.

### 2.4. Enzymatic Stability in the Presence of Commercial Detergents

The lipase stability was studied in the presence of hydrogen peroxide (0–20%) by direct incorporation into the assay mixture, and the stability was tested using several commercial laundry detergents available in the local market. For this experiment three concentrations were used (i.e., 3.3, 6.6, and 10 mg/mL) of Ariel and Ace (Procter & Gamble), Surf and Omo (Unilever), and Tixan (Ypê). Since these detergents contain some enzymes (lipases, proteases, and cellulases, according to the manufacturer), all of them were autoclaved for 30 minutes before being added to the lipase assay, to promote enzyme thermal denaturation. The same experiment was carried out using the best detergent concentration obtained at 40°C using soybean oil as substrate, for a period of 4 h. Aliquots were taken every 60 min in order to perform the enzyme assay and determine the stability.

### 2.5. Hydrolysis of Poultry Wastewater and Beef Fat Particles

Wastewater was collected from a poultry processing industry (Guapiaçu, SP, Brazil). The samples were treated with different enzyme concentrations (3–20 mg/mL) added to Tris buffer (pH 8.0), in different incubation times (0–200 min), at the lipase optimal temperature and pH obtained (40°C, pH 8.0).

Fat particles' hydrolysis experiments were conducted as described by Masse et al. [13] with some modifications. Pieces of beef fat were cut as uniform small particles (5 mm), dried at 30°C, weighed, and stored at 4°C. In 50 mL Erlenmeyer flasks, different concentrations of crude extract (5, 10, 15, and 20 mg/mL) and 100 mM Tris buffer pH 8.0 were added to one fat piece and agitated in a shaker at 30 rpm, at 40°C during 6, 12, and 24 h. The same experiment was repeated with the best enzyme concentration during 72 h in order to determine the best condition for the hydrolysis. The mixture was then filtered and the remaining particles dried at 30°C and weighed. Control samples consisted of a beef fat piece with 100 mM Tris buffer pH 8.0 under the same experimental conditions. The average particle mass data obtained during these experiments were analyzed statistically by the Student's *t*-test[26], using the freeware BioEstat 4.0 [27] and *P* < 0.05.

## 3. Results and Discussion

### 3.1. Lipase Characterization

**Figure 1:**
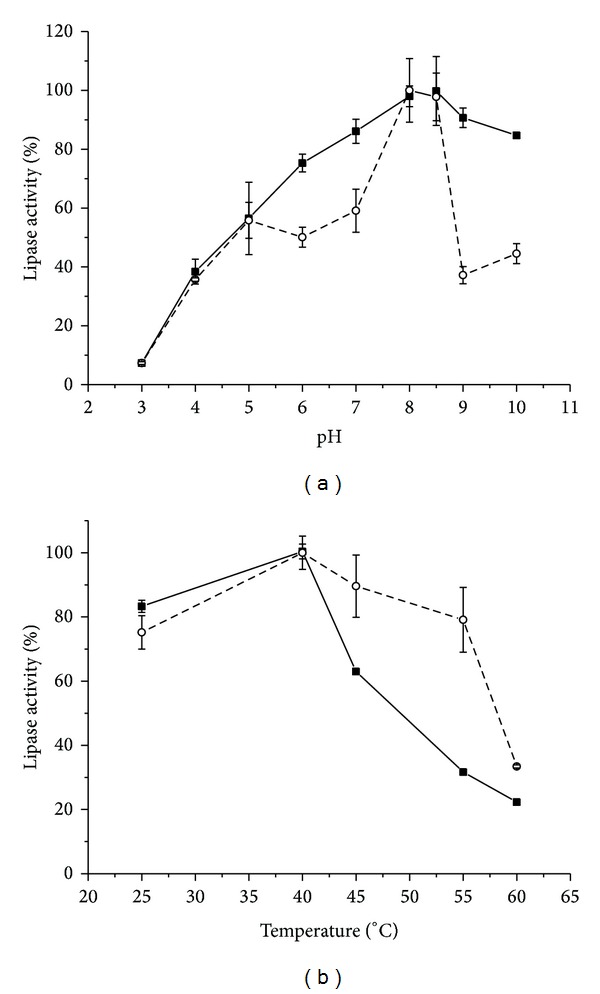
Effect of (a) pH and (b) temperature on *P. aquatica *lipase activity. Activity was measured by free fatty acids titration using soybean oil (-■-) and poultry waste water (-∘-) as enzyme substrates.

The lipase from *P. aquatica* showed higher activity in a pH range of 8.0-9.0 and at 40°C (Figures 1(a) and 1(b)) for both substrates during a 90-minute incubation period; afterwards, we observed a decrease of the hydrolysis rate, probably due to the accumulation of free fatty acids that could inhibit the enzyme [28]. Accordingly, this lipase acts at alkaline pH and mild temperatures, as those used in detergent and leather industries and wastewater treatment [5, 21, 22, 29].

These results were similar to those obtained for the synthetic substrate *p*-nitrophenyl palmitate [4]. The same optimum temperature was reported [30] for a lipase from the seeds of *Jatropha curcas* and an optimal pH of 7.5 using olive oil. The majority of studies with seed lipases indicate a neutral-alkaline optimum pH and higher activity over a temperature range from 30 to 40°C [2, 31]. However, other authors verified acid seed lipases, for example, castor bean lipase, at optimum pH values of 4.2 [32]. The optimum temperature around 40°C for this study parallels the earlier findings on lipases from coconut seeds [31], Barbados nuts [30], and rapeseeds [33].

### 3.2. Effect of Detergents and Oxidizers on Lipase Activity and Stability

**Figure 2:**
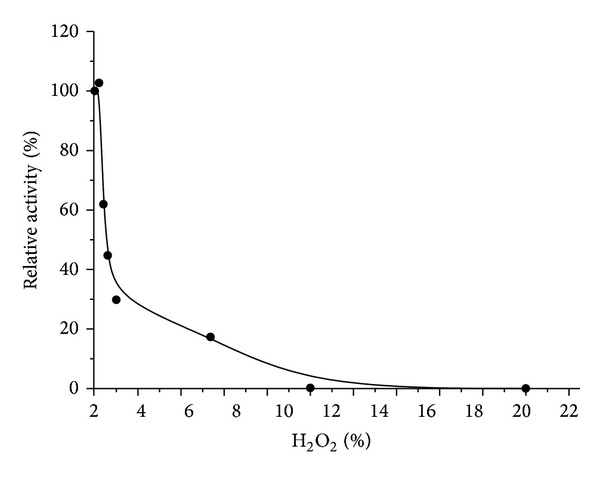
*P. aquatica* lipase activity in different concentrations of hydrogen peroxide.

In the presence of hydrogen peroxide the *P. aquatica* lipase showed a biphasic decrease of activity and retained only 30% of its activity in the presence of 1% H_2_O_2_ but still displayed 24% of the original activity at 5% H_2_O_2_ (Figure 2).

Table 1 shows that the enzyme retained approximately 100% of its activity in the presence of 3.3 mg/mL of Tixan, Surf, and Ace and around 80% using Ariel. Despite the decrease of lipase activity in Ariel and Tixan, the other detergents presented stability higher than 80% in 6.6 mg/mL. These results are very appropriate in laundry industries, since the concentration generally used in a washing machine is around 7 mg/mL [34].

**Figure 3:**
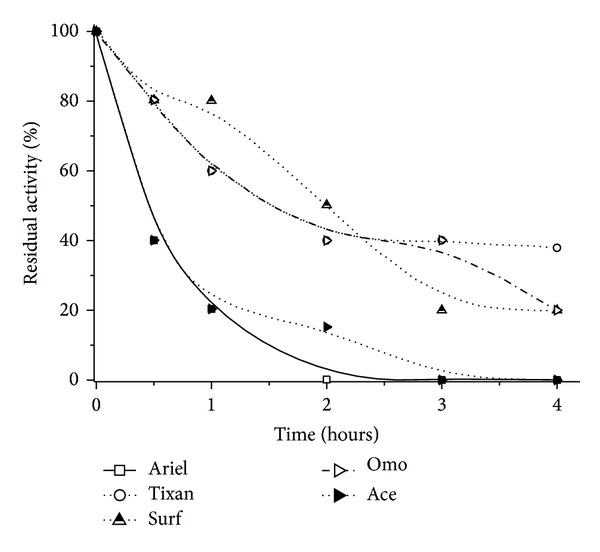
*P. aquatica *lipase stability in different commercial detergents (Ariel and Tixan—3.3 mg/mL; Surf, Ace, and Omo—6.6 mg/mL).

Another interesting result is for Omo, which obtained an increase of activity in higher detergent concentrations. Possibly, this detergent formulation could have a compound such as calcium or a tensoactive [9, 34] which increases the activity or acts at the interface water-oil, opening the enzyme active site, stabilizing its hydrophobic surface, and facilitating the hydrolysis. [35]. This stability in Omo could be easily observed in Figure 3; 40% of the lipase activity was maintained after 4 h, compared to other detergents that caused a decrease to 0–20 %.

This stability can be compared to the behavior displayed by fungal lipases. A lipase from *Aspergillus niger* showed great stability in the presence of Ariel, Surf Ultra, and Surf Excel (Saisubramanian et al., 2006 [20]), and similar results were reported for lipases from *Aspergillus* sp. and *Rhizopus* sp. [36].

### 3.3. Wastewater Hydrolysis

The concentration of free fatty acids (FFA) in wastewater was analyzed before the lipolytic treatment (0.23 ± 0.07 mM). Adding *P. aquatica* lipase to the wastewater mixture, the maximum activity was reached with 60 minutes of incubation, releasing 1.7 ± 0.35 mM FFA. Although this result represents around 10% of the total esters, it is 7.4 times higher than the concentration of the control samples. Rigo et al. [17] reported that the maximum conversion condition for a lipase from Penicillium restrictum yielded 0.1 mM of free acid/mL using 5.0% (w/v) enzyme at 45°C, in swine meat wastewater. These results are important to industry development since few studies have been published using lipases in fat wastewater. These are important results since the major constituents of animal fats are triacylglycerols consisting of saturated and unsaturated fatty acids [37].

A poultry processing plant usually generates large amounts of wastewater, both in the process itself and in the washing of equipments and facilities, well characterized by high organic and suspended solids concentration, mainly fats and protein [12]. However, the characteristics of the wastewater vary among processing plants, depending on the industrial process and the water consumed per slaughtered bird [38].

### 3.4. Fat Particles' Degradation

**Figure 4:**
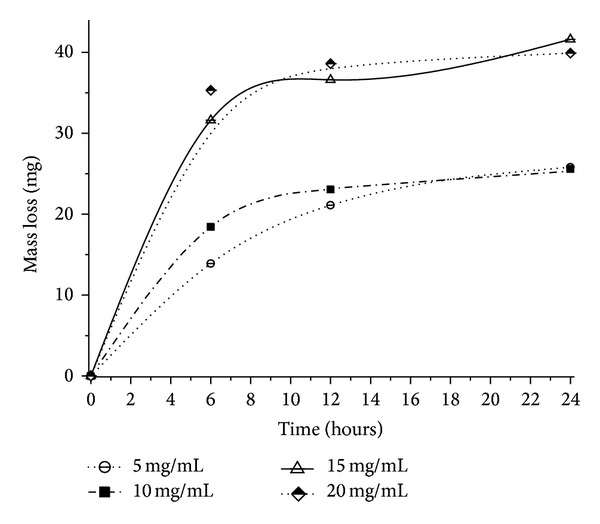
Reduction in the average mass of beef fat particles after 24 hours of pretreatment with different lipase concentrations at 40°C and pH 8.0. The initial mass was 300 mg ± 0.06.

**Figure 5:**
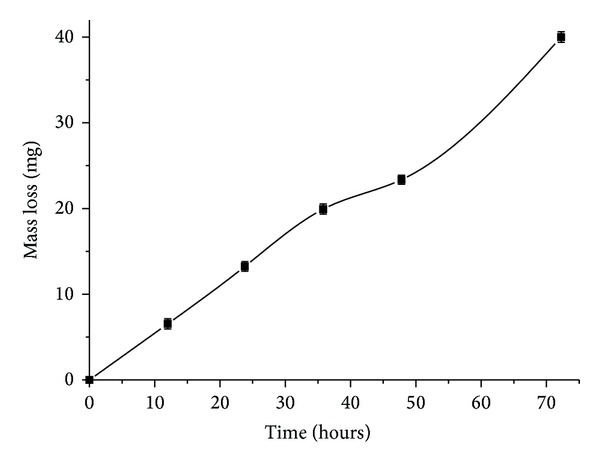
Reduction in the average mass of beef fat particles after 72 hours of pretreatment with lipase (10 mg/mL) at 40°C and pH 8.0. The initial mass was 300 mg ± 0.06.

Grindstone et al. [39] reported that, in beef, 18.4% of the triacylglycerols are composed by saturated fatty acids, while 45.1% have only one unsaturated fatty acid. In a previous study [4] it was verified that the lipase from *P. aquatica* showed higher enzymatic activity with monounsaturated oleic (18 : 1) compared to linoleic (18 : 2) and linolenic (18 : 3) acids. At this point, the effect of the lipase in fat particle degradation was tested using different enzyme concentrations and incubation time (Figures 4 and 5). After 72 hours of lipase hydrolysis, it was observed a 13% decrease in fat particle mass in solution compared to the original value. Using a higher enzyme dose (30 mg/mL), a maximum particle mass reduction was obtained (14.3%), but these results did not show significant differences when analyzed statistically. Masse et al. [13] reported no significant particle size reduction after 4 h of pretreatment with the plant lipase *EcoSystem Plus*,finding a higher effectiveness (a 40% reduction of fat particle size) using pancreatic lipase PL-250. This difference is higher once pancreatic lipase has much higher activity than plant lipases, but the major disadvantage is the difficulty to extract it. Wild type plant lipases would need a longer hydrolysis time.

## 4. Conclusions

The results in this work suggest that the crude single lipase from *P. aquatica* could be used for oil and fats wastewater pretreatment and, in future, for wastewater treatment due to its ability to hydrolyze soybean oil and other vegetable oils as shown previously [4]. The results suggest that *P. aquatica* lipase can be more effective for vegetable oil degradation than for animal fats.

## Figures and Tables

**Table 1:** Relative enzyme activity in the presence of different concentrations of commercial detergents.

Detergent	Relative activity (%)		
	3.3 mg/mL	6.6 mg/mL	10 mg/mL
Ariel	80.4 ± 0.1	60.7 ± 0.0	54.0 ± 0.1
Tixan	100.0 ± 0.1	60.5 ± 0.1	60.0 ± 0.1
Surf	100.0 ± 0.0	100.0 ± 0.1	80.0 ± 0.0
Ace	100.0 ± 0.1	100.0 ± 0.1	80.0 ± 0.1
Omo	60.0 ± 0.1	80.5 ± 0.1	80.2 ± 0.1
